# *IMPDH2*: a new gene associated with dominant juvenile-onset dystonia-tremor disorder

**DOI:** 10.1038/s41431-021-00939-1

**Published:** 2021-07-26

**Authors:** Anna Kuukasjärvi, Juan C. Landoni, Jyrki Kaukonen, Mika Juhakoski, Mari Auranen, Tommi Torkkeli, Vidya Velagapudi, Anu Suomalainen

**Affiliations:** 1 Stem Cells and Metabolism Research Program, Faculty of Medicine, University of Helsinki, Helsinki, Finland; 2 Department of Otorhinolaryngology, Mikkeli Central Hospital, Mikkeli, Finland; 3 Department of Neurology, Mikkeli Central Hospital, Mikkeli, Finland; 4 Department of Neurosciences, Helsinki University Hospital, Helsinki, Finland; 5 Metabolomics Unit, Institute for Molecular Medicine Finland (FIMM), University of Helsinki, Helsinki, Finland; 6 HUSlab, Helsinki University Hospital, Helsinki, Finland; 7 Neuroscience Center, HiLife, University of Helsinki, Helsinki, Finland

**Keywords:** Movement disorders, Disease genetics

## Abstract

The aetiology of dystonia disorders is complex, and next-generation sequencing has become a useful tool in elucidating the variable genetic background of these diseases. Here we report a deleterious heterozygous truncating variant in the inosine monophosphate dehydrogenase gene (*IMPDH2*) by whole-exome sequencing, co-segregating with a dominantly inherited dystonia-tremor disease in a large Finnish family. We show that the defect results in degradation of the gene product, causing IMPDH2 deficiency in patient cells. IMPDH2 is the first and rate-limiting enzyme in the de novo biosynthesis of guanine nucleotides, a dopamine synthetic pathway previously linked to childhood or adolescence-onset dystonia disorders. We report *IMPDH2* as a new gene to the dystonia disease entity. The evidence underlines the important link between guanine metabolism, dopamine biosynthesis and dystonia.

## Introduction

Dystonias are rare movement disorders characterised by sustained or intermittent muscle contractions causing abnormal, often repetitive, movements and/or postures. Dystonia can manifest as an isolated symptom or combined with e.g. parkinsonism or myoclonus [1]. While many pathogenic pathways are associated with dystonia, dopamine signalling is a commonly altered one [reviewed in [2]]. The first dopamine-related gene identified for dystonia was *GCH1*, encoding the rate-limiting enzyme in the pathway that converts guanosine triphosphate (GTP) to tetrahydrobiopterin (BH4) [3], which is an essential cofactor for dopamine biosynthesis (Fig. 2A). Heterozygous *GCH1* variants decrease dopamine synthesis in nigrostriatal neurons, leading to childhood-onset, progressive, dopa-responsive dystonia [4]. Variants in *HPRT1*, another purine metabolic enzyme, result in generalised dystonia with neuro-behavioural manifestations [5]. HPRT1 pathophysiology involves a guanine metabolic defect and dopamine deficiency in the midbrain, without neurodegeneration [6], underscoring the importance of dopamine metabolism for posture and movement control.

Next-generation sequencing has uncovered numerous novel dystonia genes, furthering mechanistic knowledge. However, a large portion of dystonia patients still lack a genetic diagnosis. Here, we report inosine monophosphate dehydrogenase 2 (*IMPDH2*) as a novel gene for autosomal dominantly inherited dystonia.

## Results

### Clinical description of the patients

**Fig. 1:**
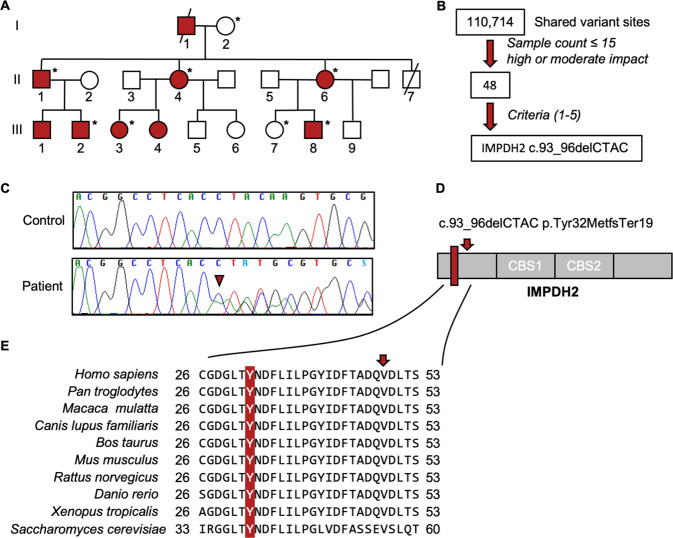
Heterozygous truncating variant in *IMPDH2* segregates in the family. **A** Pedigree of the family. Red symbols: patients. Asterisk: individuals genetically studied for *IMPDH2* variant. **B** Schematic presentation of exome data analysis and variant filtering. Variants found in 15 or less patients in our in-house database (532 patients) with an impact rating of high or moderate effect were selected for further analysis. Criteria 1–5 decribed in the Methods. **C** *IMPDH2* sequence at the deletion site (arrowhead). **D** Graphical representation of the deletion consequences for the protein. Red rectangle: deletion site; Arrow: early termination codon; CBS, cystathione-beta-synthase domains. **E** IMPDH2 protein sequence conservations at tyrosine-32, multiple sequence alignment. Arrow, location of the early stop codon.

A family of Finnish descent presented an autosomal dominantly inherited disease (Fig. 1A) characterised by dystonia and tremor. The disease-onset was between 9 and 20 years of age. Table 1 summarises the clinical presentations.

**Table 1:** Clinical presentations.

Patient	II-1	II-4	II-6	III-2	III-3	III-8
Sex	Male	Female	Female	Male	Female	Male
Age of onset	20	20	18	12	12	9
Cervical dystonia	+	+	+	+ (mild)	−	+ (mild)
Focal upper limb dystonia	+	+	+	+	+	–
Tremor (head)	Horizontal	Horizontal	Vertical	Horizontal	Horizontal	Horizontal
Tremor (hands)	Postural, action	Postural, action	postural, action	Postural, action	Postural, action	Postural
Scoliosis	Moderate	Mild	–	Mild	–	–

### Case report

Patient II-6 is a 46-year-old woman. As a teenager she experienced episodic hand and head tremor attacks with flushing, considered as panic attacks. Constant handwriting problems and muscle cramps progressively worsened from age 20. Orthopaedic and rheumatological examination (wide-spread joint-associated pain without rheumatological disease signs) led to fibromyalgia diagnosis. The patient adapted her employment because of fatigue and muscle symptoms.

At 30 years of age, she was examined due to dizziness and visual problems (struggle to focus and headache). Brain CT-scan and ophthalmological examinations were normal. The symptomatology worsened progressively from age 35, with sleeping problems, heart palpitations, limb numbness, overactive bladder, and progressive tensiogenic headache. By the age of 35, the patient had undergone three lumbar discectomies, and at age 36, she underwent a cervical discectomy. Depression was diagnosed.

At the age of 42, she was examined due to swallowing problems, and a neurological evaluation indicated cervical dystonia. Segmental dystonia in the back region as well as focal upper limb dystonia were suspected. She had essential hand tremor and tremor in the head and upper back. Walking, speaking, balance, and eye movements were normal. Sensory testing showed distal lower leg allodynia and signs of hyperalgesia. Cold hypaesthesia was evident in both legs (distal from the right ankle and left knee) and cold hyperaesthesia on the right shin. Brain MRI and DAT scans were normal, and spinal MRI showed cervical and lumbar degeneration without any nerve contact or medullopathy. Bone scintigraphy was normal. Electromyoneurography was normal, but histological analysis of a skin sample from the lower leg diagnosed clear small fibre neuropathy with no observed subcutaneous nerve fibres.

### Whole-exome sequencing identifies a heterozygous *IMPDH2* variant

We sequenced the exomes of three patients (II-1, II-6 and III-3). The identified shared variants were assessed based on rarity, predicted severity, conservation and gene function (Fig. 1B), yielding a single candidate gene, *IMPDH2*. A heterozygous four-base deletion led to an early termination in the first exon of *IMPDH2* (NM_000884.2:c.93_96del p.(Tyr32MetfsTer19), NC_000003.12:g. 49066688_49066691del) (Fig. 1D). No homozygotes for the variant allele and two heterozygous carriers of Finnish descent, potential undiagnosed or presymptomatic subjects, were found in the gnomAD database (http://gnomad.broadinstitute.org/) making the variant extremely rare. Multiple sequence alignment showed a high conservation of IMPDH2 from human to yeast (Fig. 1E). Sanger sequencing of samples from six affected family members and two nonaffected family members confirmed the complete co-segregation of the dominant variant in the dystonic subjects (Fig. 1A, C).

### Mutant *IMPDH2* transcript is degraded leading to an IMPDH2 deficiency

**Fig. 2:**
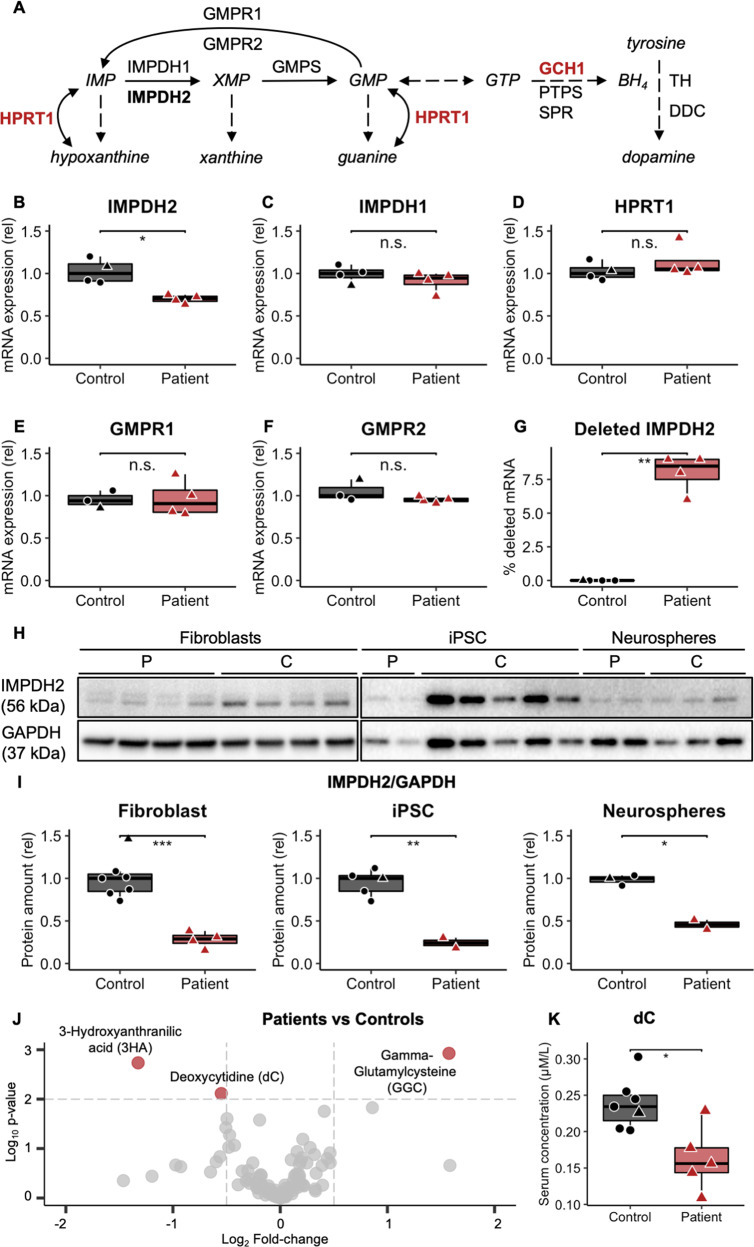
*IMPDH2* deletion transcript leads to IMPDH deficiency. Red: patients. Black: controls. Healthy family member: black triangle. **A** IMPDH2: rate-limiting enzyme and first reaction in de novo biosynthesis of guanine nucleotides. Enzymes previously associated with dystonia in red. BH tetrahydrobiopterin, DDC aromatic amino acid decarboxylase, GCH1 GTP cyclohydrolase I, GMPR guanosine monophosphate reductase, GMPS guanosine monophosphate synthetase, HPRT1 hypoxanthine phosphoribosyltransferase 1, PTPS pyruvoyl-tetrahydropterin synthase, SPR sepiapterin reductase, TH tyrosine hydroxylase. **B**–**F** Relative (rel) mRNA expression of *IMPDH2*, *IMPDH1, HPRT1, GMPR1* and *GMPR2*. **G** Quantification of *IMPDH2* transcript with deletion in patients and controls. **H**, **I** IMPDH2 protein amount in fibroblasts, induced pluripotent stem cells (iPSC) and differentiated neurospheres. **J** Volcano plot: serum metabolic profile changes between patients and healthy controls. In red: significantly changed metabolites (Fold-change > 1.5 and *p* value < 0.01). **K** Deoxycytidine (dC) concentration in serum. Statistical significance: pairwise two-tailed *t* test (*p ≤ 0.05, **p ≤ 0.01, ***p ≤ 0.001).

IMPDH2 is the first and rate-limiting enzyme in de novo GMP biosynthesis, oxidising inosine monophosphate into xanthosine monophosphate [7] (Fig. 2A).

Cultured fibroblasts showed a reduced *IMPDH2* transcript amount in patients, with ~70% of residual mRNA (Fig. 2B), from which ~10% is mutant mRNA (Fig. 2G). The finding indicates almost complete degradation of the mutant transcript, consistent with nonsense-mediated mRNA decay due to the early stop codon.

To study a physiologically relevant cell type, we generated induced pluripotent stem cells (iPSCs) and induced them to a neural lineage. The total protein amount of IMPDH2 and its highly homologous isoform IMPDH1 were detected together, as specific antibodies against IMPDH2 are unavailable to our knowledge [8]. The isoforms likely can heterotetramerize, but show different expression patterns, *IMPDH2* being the major form in the central nervous system (CNS).

Total IMPDH protein was decreased down to 70% in the patient fibroblasts, iPSCs and neurospheres compared to controls (Fig. 2H, I). The reduction was more severe than expected, as *IMPDH2* mRNA was reduced by 30% and *IMPDH1* mRNA expression was control-like (Fig. 2C). The expression of the enzymes participating in alternative or *IMPDH2-*related pathways were unchanged (Fig. 2D–F). The *IMPDH2* variant leads to depletion of the total protein pool of IMPDH, suggesting that IMPDH2 amount and its metabolic consequences regulate protein stability of both IMPDH2 and IMPDH1. These findings suggest that IMPDH2 depletion causes reduction of both IMPDH isoforms post-transcriptionally.

### Patients present normal serum metabolomic profile and nucleotide balance

The serum metabolomic analysis of five patients, one unaffected family member and five age and gender-matched controls was performed by a semi-quantitative targeted panel of 102 metabolites. Three significantly changed metabolites were found (Fig. 2J), out of which two were most enriched in the family members independent of the disease status (Fig. S1). Deoxycytidine, a nucleotide precursor, was reduced in the patients (Fig. 2K), suggesting nucleotide metabolic disbalance. In fibroblasts, our quantitative deoxynucleoside triphosphate (dNTP) pool concentration analysis showed normal dNTP pools in dividing and quiescent fibroblasts (Figs. S2, and S3).

## Discussion

Here we report *IMPDH2* as a novel disease gene for dominantly inherited juvenile-onset dystonia-tremor disorder. The causative role of *IMPDH2* was supported by (1) the complete segregation and penetrance of the manifestation in a large pedigree; (2) high conservation of the protein and mutation site in species; (3) remarkably decreased gene product and (4) pathomechanistic similarity to previously reported dopamine-linked dystonia pathways. Furthermore, a recent large study focusing on neurodevelopmental disorders with dystonia raised attention to *IMPDH2* as a candidate gene [9], but direct evidence has been lacking.

IMPDH2 and IMPDH1 are tissue-specific enzyme isoforms, rate-limiting in the de novo guanine biosynthesis pathway. *IMPDH1* variants underlie autosomal dominant retinopathy [10, 11], but *IMPDH2* had not yet been assigned to a disease. We found a heterozygous truncating variant of *IMPDH2* to decrease protein levels of both IMPDHs, more severely than predicted by mRNA levels. The finding suggests that low IMPDH2 amount or involved metabolites signal for high guanine, downregulating IMPDH1 as well as preventing compensatory GTP-synthetic pathways. We propose that in post-mitotic cells of the CNS, with considerably lower nucleotide pools than in cultured fibroblasts [12] and high IMPDH2 expression, the enzyme defect becomes rate-limiting, challenging guanine and dopamine synthesis and resulting in dystonia and tremor.

Clinically, IMPDH2-linked dystonia mimicks other dominantly inherited dystonias such as those caused by variants in *GCH1*, also an enzyme in the GTP-BH4 pathway. The IMPDH2 reaction is flanked by HPRT1 products—also a dystonia-linked protein—upstream from GCH1. The conversion of GTP into the dopamine biosynthetic cofactor BH4 is often affected in genetic dystonias (Fig. 2A) and also linked to pain sensitivity [13]. One of our patients showed sensory defects in the legs, whether this is linked to BH4 remains to be shown. A heterozygous variant in *GMPR* (GMP reductase), the enzyme catalysing the reverse reaction of IMPDH2, causes autosomal dominant progressive external ophthalmoplegia with muscle mitochondrial respiratory chain defect [14]. Our findings highlight the cell-type-specific importance of metabolic pathways and their directionality and point towards a shared pathophysiological mechanism behind *IMPDH2* deficiency and other inherited dystonias.

In conclusion, *IMPDH2* is a novel dystonia gene linked to the dopamine synthesis pathway, implying that the symptoms may be L-DOPA responsive. Improved genetic knowledge is highly valuable for diagnosis and therapy choices for this complex and heterogeneous disease group.

Materials and methods are described in the supplementary information.

## Supplementary information

Supplemental Materials and Methods

Supplementary Table 1.

Supplementary Tables 2-3.

Supplementary figures 1–3.

Supplementary figures 4-7.

## References

[1] Albanese A, Bhatia K, Bressman SB, Delong MR, Fahn S, Fung VSC (2013). Phenomenology and classification of dystonia: a consensus update. Mov Disord Mov Disord.

[2] Jinnah HA, Sun YV. Dystonia genes and their biological pathways. Neurobiol Dis. 2019;129:159–68. 10.1016/j.nbd.2019.05.014 31112762

[3] Nichol CA, Smith GK, Duch DS (1985). Biosynthesis and metabolism of tetrahydrobiopterin and molybdopterin. Annu Rev Biochem..

[4] Ichinose H, Ohye T, Takahashi E, Seki N, Hori T, Segawa M (1994). Hereditary progressive dystonia with marked diurnal fluctuation caused by mutations in the GTP cyclohydrolase I gene. Nat Genet..

[5] Lesch M, Nyhan WL (1964). A familial disorder of uric acid metabolism and central nervous system function. Am J Med..

[6] Göttle M, Prudente CN, Fu R, Sutcliffe D, Pang H, Cooper D (2014). Loss of dopamine phenotype among midbrain neurons in Lesch-Nyhan disease. Ann Neurol.

[7] Magasanik B, Moyed HS, Gehring LB (1957). Enzymes essential for the biosynthesis of nucleic acid guanine; inosine 5’-phosphate dehydrogenase of Aerobacter aerogenes. J Biol Chem..

[8] Natsumeda Y, Ohno S, Kawasaki H, Konno Y, Weber G, Suzuki K (1990). Two distinct cDNAs for human IMP dehydrogenase. J Biol Chem..

[9] Zech M, Jech R, Boesch S, Škorvánek M, Weber S, Wagner M (2020). Monogenic variants in dystonia: an exome-wide sequencing study. Lancet Neurol..

[10] Bowne SJ, Sullivan LS, Blanton SH, Cepko CL, Blackshaw S, Birch DG (2002). Mutations in the inosine monophosphate dehydrogenase 1 gene (IMPDH1) cause the RP10 form of autosomal dominant retinitis pigmentosa. Hum Mol Genet..

[11] Kennan A, Aherne A, Palfi A, Humphries M, McKee A, Stitt A (2002). Identification of an IMPDH1 mutation in autosomal dominant retinitis pigmentosa (RP10) revealed following comparative microarray analysis of transcripts derived from retinas of wild-type and Rho(-/-) mice. Hum Mol Genet..

[12] Gandhi VV, Samuels DC (2011). A review comparing deoxyribonucleoside triphosphate (dNTP) concentrations in the mitochondrial and cytoplasmic compartments of normal and transformed cells. Nucleosides Nucleotides Nucleic Acids..

[13] Latremoliere A, Latini A, Andrews N, Cronin SJ, Fujita M, Gorska K (2015). Reduction of neuropathic and inflammatory pain through inhibition of the tetrahydrobiopterin pathway. Neuron..

[14] Sommerville EW, Dalla Rosa I, Rosenberg MM, Bruni F, Thompson K, Rocha M (2020). Identification of a novel heterozygous guanosine monophosphate reductase (*GMPR*) variant in a patient with a late onset disorder of mitochondrial DNA maintenance. Clin Genet..

