# Supplementary material for: IMPDH2: a new gene associated with dominant juvenile-onset dystonia-tremor disorder
Source: Eur J Hum Genet. 2021 Jul 26;29(12):1833–7. doi: 10.1038/s41431-021-00939-1 (PMC8633184; doi:10.1038/s41431-021-00939-1)
Supplement: Supplementary file 1 — Supplemental Materials and Methods [file 41431_2021_939_MOESM1_ESM.docx]

# **Materials and Methods**

## **Ethical protocols**

## All samples were collected according to the Declaration of Helsinki, with informed consent from the subjects. The ethical permit number is 325/13/03/00/2015.

## **Whole exome sequencing**

The patients’ genomic DNA was extracted from whole blood using standard procedures. Whole-Exome sequencing was performed using NimbleGen Sequence Capture 2.1M Human Exome V.2.0. The variant calling pipeline from our laboratory and the Institute of Molecular Medicine Finland (FIMM, Helsinki) (1,2) was used for alignment and variant identification, using GRCh37 as the reference genome. The variants were filtered to identify those shared by the three sequenced patients, found in 15 or less patients in our in-house database of 532 patients and presenting a high or moderate impact rating, based on annotation by Ensembl Variant Effect Predictor (<https://www.ensembl.org/vep>). The resulting variants are listed in Supplementary material Table 1. The resulting variants were not prioritized if:

1. Variant in an intronic, noncoding, non-functional, or untranslated region of the genome.
2. Homozygotes or allele count of heterozygotes ≥ 25 in population databases [gnomAD [(gnomad.broadinstitute.org](file:///Users/aewartio/Library/Containers/com.apple.mail/Data/Library/Mail%20Downloads/9544AF70-BEE5-414F-BC8C-7E6D584876C4/(gnomad.broadinstitute.org)), ExAC (now part of gnomAD) and MitoMAP ([mitomap.org/MITOMAP](https://www.mitomap.org/MITOMAP))].
3. Variant found in in-house database, associated with a different phenotype.
4. Low amino acid sequence conservation across species.
5. No appropriate protein function-phenotype relationship for a CNS disorder.

## **DNA sequence analysis**

Sanger sequencing was performed using standard measures, from six affected family members (II-1, II-4, II-6, III-2, III-3, III-8) and two nonaffected family members (I-2, III-7). Sequences were analysed using Sequencher® ([genecodes.com](http://www.genecodes.com/%22%20/)). The primers used are listed in the Supplementary material Table 2.

## **Fibroblast culture**

The primary skin fibroblasts from subjects were cultured in fibroblast culture medium [DMEM with 10% FBS, penicillin/streptomycin and GlutaMAX^TM^ supplementation (Gibco^TM^ Dublin, Ireland)].

**iPSC generation and neurosphere differentiation**

iPSC lines (2 patient lines, 1 line from a healthy family member, 2 control lines with two clones from each) were generated from dermal fibroblasts as described in (3). The cells were electroporated with three episomal plasmids [27077: pCXLE-hOCT3/4-shp53-F, 27078: pCXLE-hSK, 27079: pCXLE-hMLN; Addgene (Watertown, MA, USA)] following the manufacturer’s instructions [Neon Transfection system: Invitrogen; Life Technologies (Carlsbad, CA, USA)]. Cells were cultured for a week in Fibroblast medium and plated in low-density, switching to hES medium [DMEM/F12 + GlutaMAX^TM^ Life Technologies 31331-028, KO-Serum Replacement Gibco 10828-010, Non-Essential Amino-Acids 100x: Gibco 11140-035, 2-β-Mercaptoethanol 14.3M, Merck M3148 (Darmstadt, Germany), Sodium Butyrate 500mM Merck TR-1008-G, Human bFGF 40µg/ml Peprotech 100-18B (Rocky Hill, NJ, USA), Uridine 500x (Merck 6680)]. Colonies were mechanically picked after 20-30 days on Matrigel plates [Corning 354277 (Corning, NY, USA)] with hES media, and E8 medium after 2 passages (Essential 8 medium: Life Technologies: A1517001) on Matrigel-coated plates. Silencing of episomal vectors was confirmed by PCR.

Neural differentiation (2 patient cell lines, 1 healthy family member, 1 control line with two clones) was performed as described in (4). In short, iPSCs were plated with Rho kinase inhibitor on low-attachment plates (Corning) in neural differentiation medium [DMEM/F-12 (Gibco 31330-038) 96, Neurobasal (Gibco 21103-049), GlutaMAX (Gibco 35050-038), B27 without vitamin A (Gibco 12587-010), N2 (Gibco 17502-048), penicillin/streptomycin (Gibco 15070-063), 20 ng/ml bFGF)]. The neurospheres formed were expanded by manually by cutting, to maintain a sphere diameter of 500-700 µm, and collected after 5 weeks of culture.

## **qPCR and cDNA sequencing**

RNA was extracted from fibroblasts using TRIzol Reagent (Invitrogen #15596018).

cDNA was synthesised (Maxima First Strand cDNA Synthesis kit with dsDNAse; Thermo Fisher Scientific #K1672, Waltham, MA, USA) and qPCR performed (2x SensiFAST SYBR No-ROX Mix;Thermo Fisher Scientific #BIO-98020). List of the PCR primers: Supplementary material Table 3.

IMPDH2 cDNA was Sanger-sequenced with primers: 5’-CCATGGCCGACTACCTGATT-3’ (forward) and 5´-CGAACTTCATTGGCCTGGAA-3’ (reverse). The results were quantified using the ICE software (ice.synthego.com).

## **Western blot**

Total protein extracts were prepared from fibroblasts, iPSC and neurospheres using RIPA buffer containing protease + phosphatase inhibitor (Thermo Fisher Scientific A32955 and 78444). Protein concentration was quantified (Pierce BCA Protein Assay Kit; Thermo Fisher Scientific 23227) and 7.5 micrograms of protein was analysed by Western blot, using standard methods and following antibodies: IMPDH2: Abcam, #ARP54365_P05; GAPDH: Cell Signaling Technology, #2118S. Images of uncropped blots: Supplementary figures 4-7.

## **Serum metabolomics**

Serum of five patients (II-1, II-4, II-6, III-2, III-8), one unaffected family member (III-7) and five age and gender-matched controls were collected in the morning after ten hours of fasting. Targeted metabolomic analysis was carried out at the FIMM metabolomics core facility as described previously (5).

## **dNTP pools**

Quantification of deoxynucleoside triphosphate pools and concentrations of each canonical dNTP was done by solid-phase radio-labelled polymerase incorporation assay, as described in (6), and measured by a scintillation counter. The concentrations were determined from a standard curve.

**References**

1. Sulonen A-M, Ellonen P, Almusa H, Lepistö M, Eldfors S, Hannula S, et al. Comparison of solution-based exome capture methods for next generation sequencing. Genome Biol [Internet]. 2011 [cited 2019 Dec 1];12(9):R94. Available from: http://genomebiology.biomedcentral.com/articles/10.1186/gb-2011-12-9-r94

2. Carroll CJ, Brilhante V, Suomalainen A. Next-generation sequencing for mitochondrial disorders. Br J Pharmacol [Internet]. 2014 [cited 2019 Jun 6];171(8):1837–53. Available from: https://www.ncbi.nlm.nih.gov/pmc/articles/PMC3976608/

3. Hämäläinen RH, Suomalainen A. Generation and characterization of induced pluripotent stem cells from patients with mtDNA mutations. In: Methods in Molecular Biology [Internet]. Humana Press Inc.; 2016 [cited 2021 Feb 3]. p. 65–75. Available from: https://link.springer.com/protocol/10.1007/7651_2015_258

4. Lappalainen RS, Salomäki M, Ylä-Outinen L, Heikkilä TJ, Hyttinen JA, Pihlajamäki H, et al. Similarly derived and cultured hESC lines show variation in their developmental potential towards neuronal cells in long-term culture. Regen Med [Internet]. 2010 Sep 27 [cited 2020 Sep 10];5(5):749–62. Available from: https://www.futuremedicine.com/doi/abs/10.2217/rme.10.58

5. Nandania J, Peddinti G, Pessia A, Kokkonen M, Velagapudi V. Validation and Automation of a High-Throughput Multitargeted Method for Semiquantification of Endogenous Metabolites from Different Biological Matrices Using Tandem Mass Spectrometry. Metabolites [Internet]. 2018 Aug 5 [cited 2019 Dec 1];8(3):44. Available from: http://www.mdpi.com/2218-1989/8/3/44

6. Landoni JC, Wang L, Suomalainen A. Quantitative solid-phase assay to measure deoxynucleoside triphosphate pools. Biol Methods Protoc [Internet]. 2018 Jan 1 [cited 2018 Oct 14];3(1). Available from: https://academic.oup.com/biomethods/article/doi/10.1093/biomethods/bpy011/5127789
