# Supplementary figures and images for: IMPDH2: a new gene associated with dominant juvenile-onset dystonia-tremor disorder
Source: Eur J Hum Genet. 2021 Jul 26;29(12):1833–7. doi: 10.1038/s41431-021-00939-1 (PMC8633184; doi:10.1038/s41431-021-00939-1)

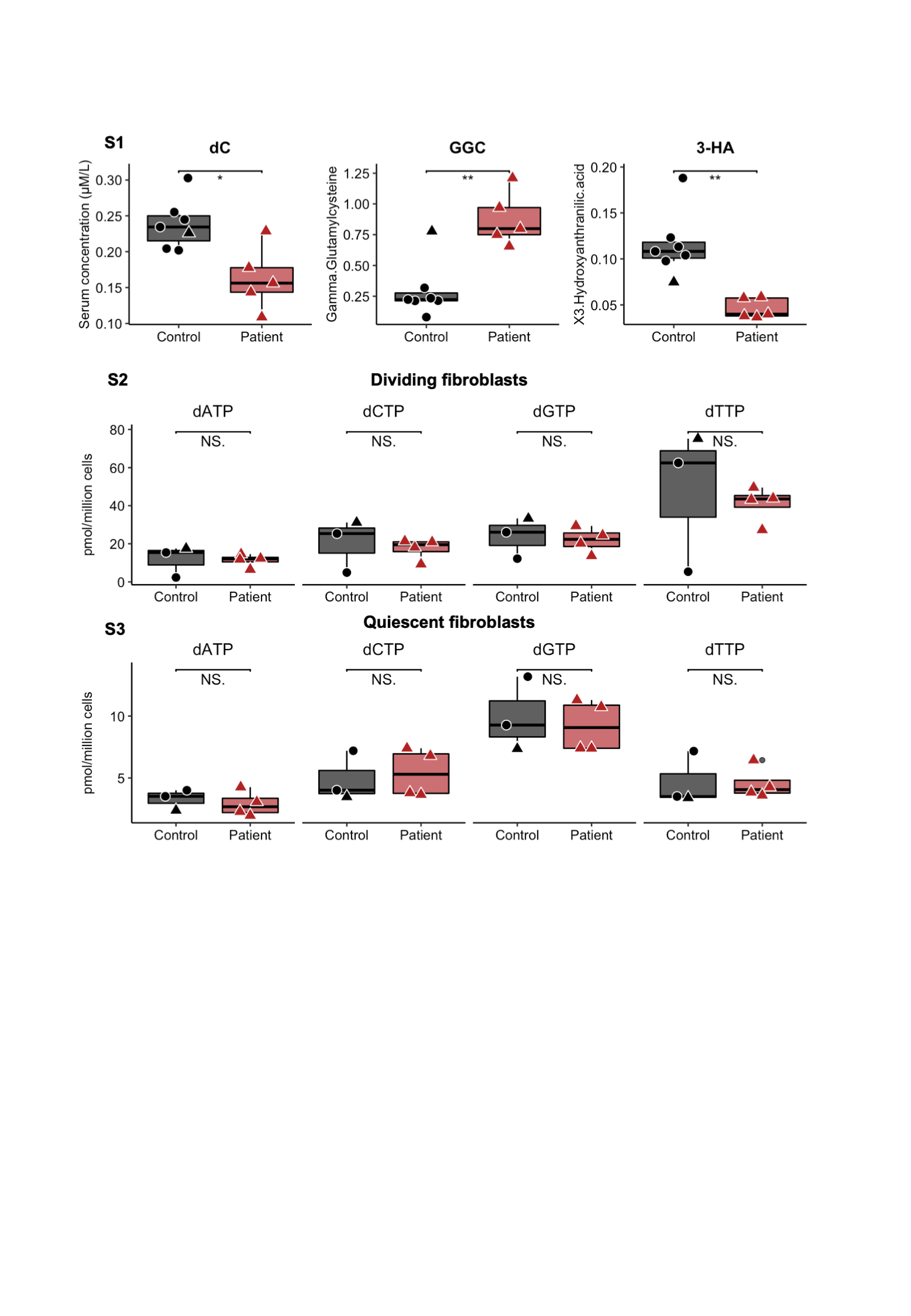

Supplement: Supplementary file 4 — Supplementary figures 1–3. [file 41431_2021_939_MOESM4_ESM.tif]

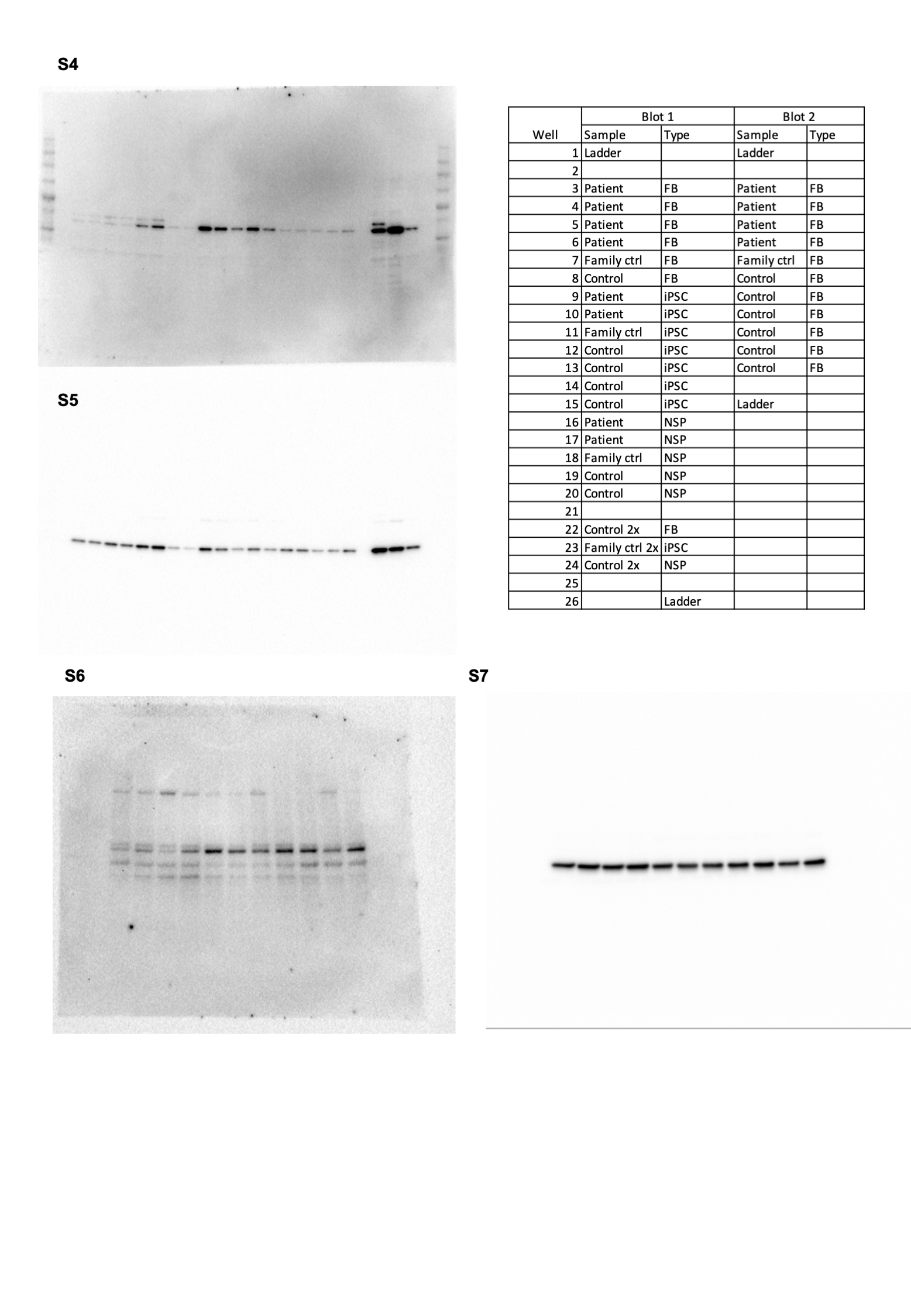

Supplement: Supplementary file 5 — Supplementary figures 4-7. [file 41431_2021_939_MOESM5_ESM.tif]
